# Supplementary material for: The RNA polymerase II subunit RPB‐9 recruits the integrator complex to terminate Caenorhabditis elegans piRNA transcription
Source: EMBO J. 2021 Feb 3;40(5):e105565. doi: 10.15252/embj.2020105565 (PMC7917558; doi:10.15252/embj.2020105565)
Supplement: Supplementary file 1 — Appendix [file EMBJ-40-e105565-s001.pdf]

Table of Contents

Appendix Figure S1. Inheritance of silencing 2

Appendix Figure S2. Differential expression of genes in total RNA libraries 3

Appendix Figure S3. Cross between *rpb-9* and *tfiis* mutants..4

Appendix Table S1. *C. elegans* strains...5

Appendix Table S2. Genotyping primers, CRISPR repair template and guide RNAs 6

Appendix Table S3. qPCR primers...7

Percentage of desilenced animals

	**wild type**					***rpb-9***					***hrde-1***					***prg-1; prg-2***				
	r1	r2	r3	r4	r5	r1	r2	r3	r4	r5	r1	r2	r3	r4	r5	r1	r2	r3	r4	r5
**P0**	0	0	0	0	0	0	0	0	0	0	0	0	0	0	0	0	0	0	0	0
**G1**	0	0	0	0	0	0	0	0	0	0	100	100	100	100	100	0	0	0	0	0
**G2**	0	0	0	0	0	0	0	0	0	0	100	100	100	100	100	0	0	0	0	0
**G3**	0	0	0	0	0	0	0	0	0	0	100	100	100	100	100	0	0	0	0	0
**G4**	0	0	0	0	0	0	0	0	0	0	100	100	100	100	100	0	0	0	0	0
**G5**	0	0	0	5.33	0	0	0	5.12	St	0	100	100	100	100	100	0	0	2.34	0	0
**G6**	0	3.23	0	10.65	6.75	9	8.73	St		8.76	100	100	100	100	100	4.34	6.75	5.26	12.76	7.78

Appendix Figure S1. **Related to Figure 2. A**) Table of the inheritance of silencing experiment, showing the percentages of desilenced animals at each generation for individual replicates separately, for all the indicated genotypes.

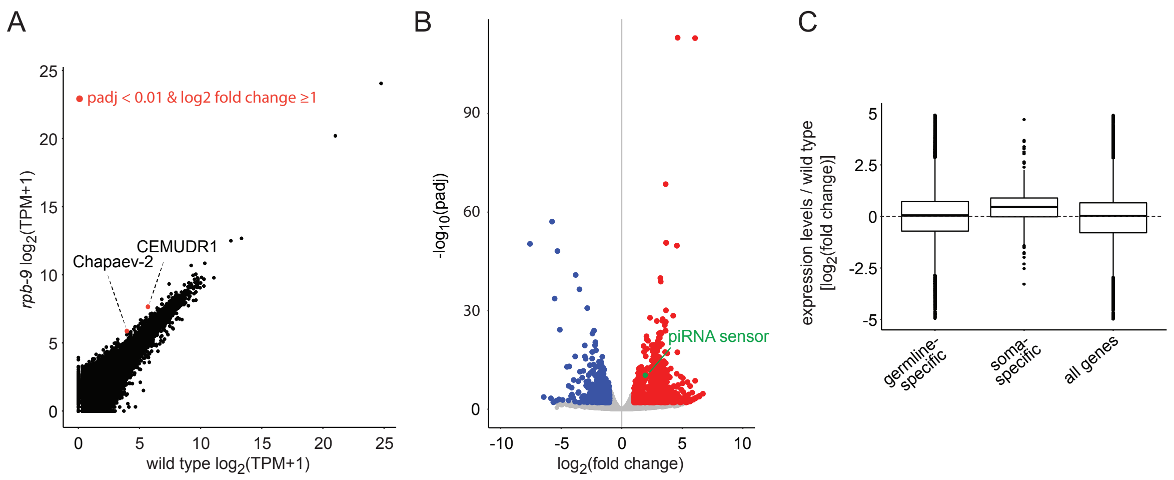

Appendix Figure S2. **Related to Figure 3. A**) Differential expression analysis of transposable elements in *rpb-9 (mj261)* mutants versus wild type (total RNA “Ribo-Zero” RNA-seq libraries). **B**) Genome-wide differential expression analysis of *rpb-9 (mj261)* mutants versus wild type (total RNA “Ribo-Zero” RNA-seq libraries). **C**) Differential expression analysis of germline-specific and soma-specific genes, as classified in (Reinke et al, 2004). All genes are shown for comparison. (Total RNA “Ribo-Zero” RNA-seq libraries).

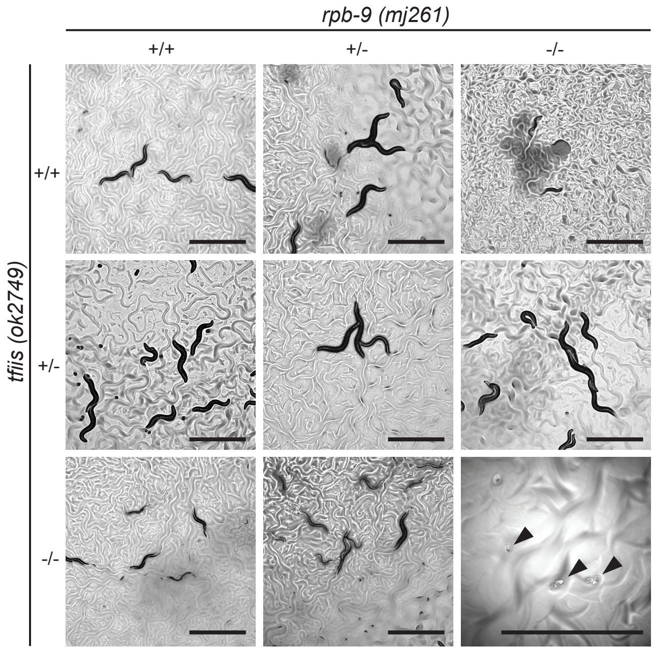

Appendix Figure S3. **Cross between *rpb-9* and *tfiis* mutants.** Representative bright light images of all the genotypes obtained from the *rpb-9 (mj261)* x *tfiis (ok2749)* cross (F3 generation). *Tfiis; rpb-9* double mutant embryos (indicated with black arrowheads) do not hatch. Scale bar = 1mm.

C. elegans: SX1316 – *piRNA sensor (mjIs144)*	Ashe et al., 2012
C. elegans: SX1984 – *rpb-9 (mj261)*	This paper
C. elegans: SX3501 – *(mjIs31); rpb-9 (mj261)*	This paper
C. elegans: SX2646 [rescue] – *(mjSi70); rpb-9 (mj261)*	This paper, injections of EG6701, cross SX1984
C. elegans: SX3031 [deficient rescue] – *(mjSi89); sensor (mjIs144); rpb-9 (mj261)*	This paper, injections of EG6701, cross SX1316 and SX1984
C. elegans: VC20618 – *rpb-9* (*gk357431*)	(Thompson et al. 2013)
C. elegans: VC40196 – *rpb-9* (*gk508135*)	(Thompson et al. 2013)
C. elegans: SX3317 [*rpb-9::ollas*] – *rpb-9 (mj604)*	This paper, injections N2 strain
C. elegans: SX2000 – *hrde-1 (tm1200); (mjIs144)*	Ashe et al., 2012
C. elegans: SX1888 – *prg-1 (n4357); (mjIs144)*	Ashe et al., 2012
C. elegans: FX1200 – *hrde-1 (tm1200)*	Buckley et al., 2012
C. elegans: SX1158 – *prg-1 (n4357)*; (*mjIs144); prg-2 (n4358)*	Ashe et al., 2012
C. elegans: SX3376 – *sensor (mjIs144); rpb-9 (mj604)*	This paper, cross SX3317 x SX1316

Appendix Table S1. *C. elegans* strains used in this study.

rpb-9 (mj261) genotyping primer fwd: CCCGAAGACTGAAGAACATC
rpb-9 (mj261) genotyping primer rev: TATTGGAACAGGTAGGGAACA
rpb-9::ollas (mj604) genotyping primer fwd: CTGTGAGCATCGTGAAGT
rpb-9::ollas (mj604) genotyping primer rev:<br>AGGTAGGGAACATATGGAGA
RPB9::ollas CRISPR HR template:<br>GGCAGAAGAAGAGATGAGATTGTACTATGTATGTGCATCGCAAGATTGTCAGCACAGATGGACaGAAGGAGGTGGAG<br>GTGGAGCTTCCGGATTCGCCAACGAGCTCGGACCACGTCTCATGGGAAAGAtaaACATAGTGTCCAATTGTTTTCTATTT<br>ATTTGAAACGTTTTCTTAATCGTC
RPB-9::ollas CRISPR gRNA: CGCAAGAUUGUCAGCACAGA
dpy-10 CRISPR gRNA:<br>GCUACCAUAGGCACCACGAG

Appendix Table S2. Genotyping primers, CRISPR repair template and guide RNAs used in this study.

Spliced gfp ex1 fwd: GTGAAGGTGATGCAACATACGG
Spliced gfp ex1-ex2 rev: ACAAGTGTTGGCCATGGAAC
Unspliced gfp ex1 fwd: AACATACGGAAAACTTACCCTTA
Unspliced gfp ex2-in1 rev: CAAGTGTTGGCTGAAAATTTAAA
Unspliced gfp ex2-in2 fwd: TGACGGGAACTACAAGACACG
Unspliced gfp ex3-in2 rev: TCAAACTTGACTTCAGCACCTG
Unspliced gfp ex3 fwd: ACAATGTATACATCATGGCAGAC
Unspliced gfp ex4-in3 rev: CTAATTTTGAAGTTCTGAAAATT
cdc-42 for: CTGCTGGACAGGAAGATTACG
cdc-42 rev: CTCGGACATTCTCGAATGAAG

Appendix Table S3. qPCR primers used in this study.
