# Supplementary material for: The RNA polymerase II subunit RPB‐9 recruits the integrator complex to terminate Caenorhabditis elegans piRNA transcription
Source: EMBO J. 2021 Feb 3;40(5):e105565. doi: 10.15252/embj.2020105565 (PMC7917558; doi:10.15252/embj.2020105565)
Supplement: Supplementary file 2 — Expanded View Figures PDF [file EMBJ-40-e105565-s002.pdf]

# Expanded View Figures

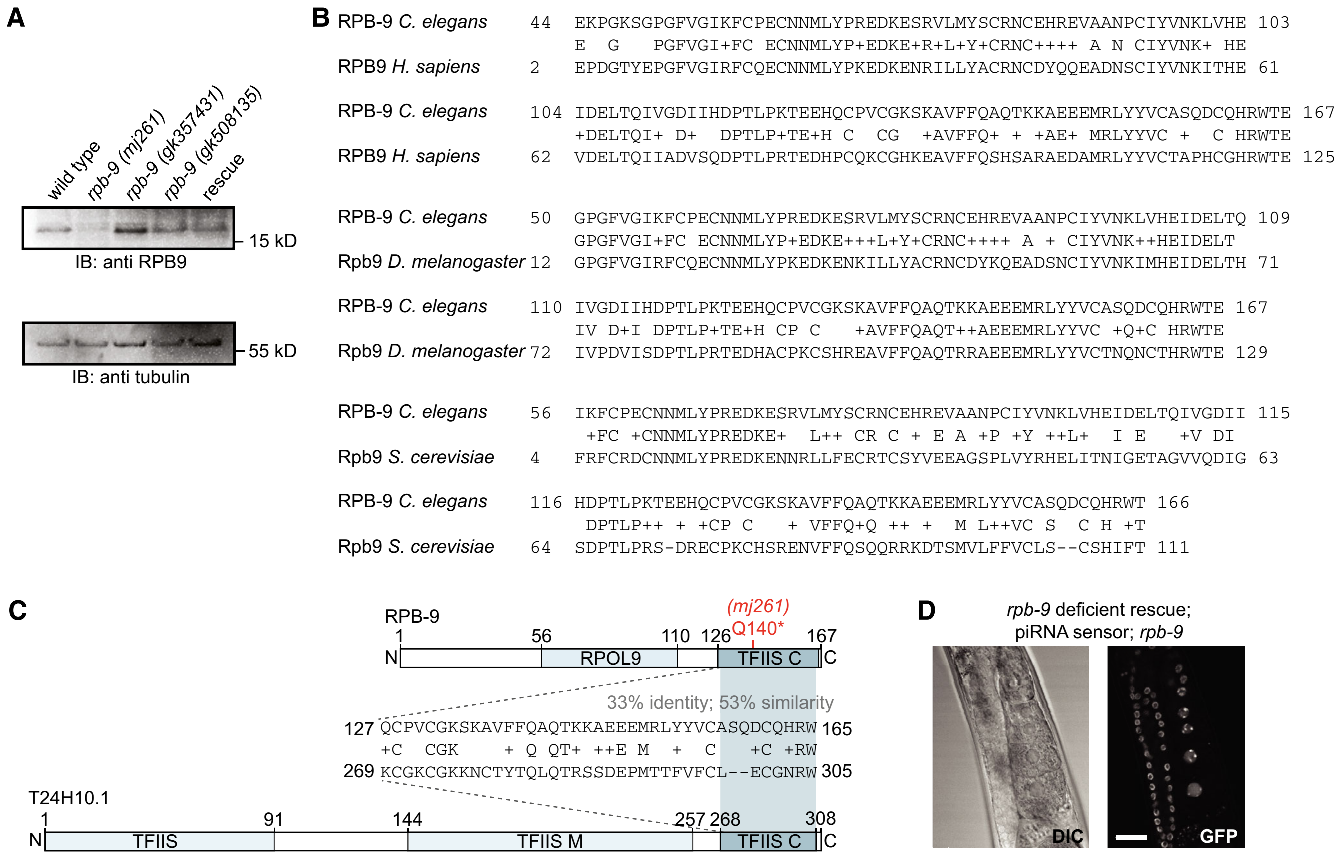


**Figure EV1. Related to Fig 1.**

A Western blot quantification of RPB-9 expression in wild-type, *rpb-9 (mj261)*, and *rpb-9* rescue animals. *rpb-9* alleles *gk508135* and *gk357431* (Thompson *et al*, 2013) are additional controls that do not show piRNA sensor desilencing.

B RPB-9 domain conservations. Alignments of the RPB-9 protein to its *H. sapiens*, *D. melanogaster* and *S. cerevisiae* homologs. Positive (NCBI BlastP) amino acids are indicated by "+".

C Comparison of RPB-9 and the *Caenorhabditis elegans* TFIIS homolog T24H10.1 with alignment of the common TFIIS C domain. The introduced RPB-9 Q140* mutation is highlighted in red. Positive (NCBI BlastP) amino acids are indicated by "+".

D Representative DIC and fluorescence microscopy images of piRNA sensor expression in deficient rescue *rpb-9 (mj261; mjSi89)* animals. Scale bar = 20 µm.

Source data are available online for this figure.


© 2021 The Authors

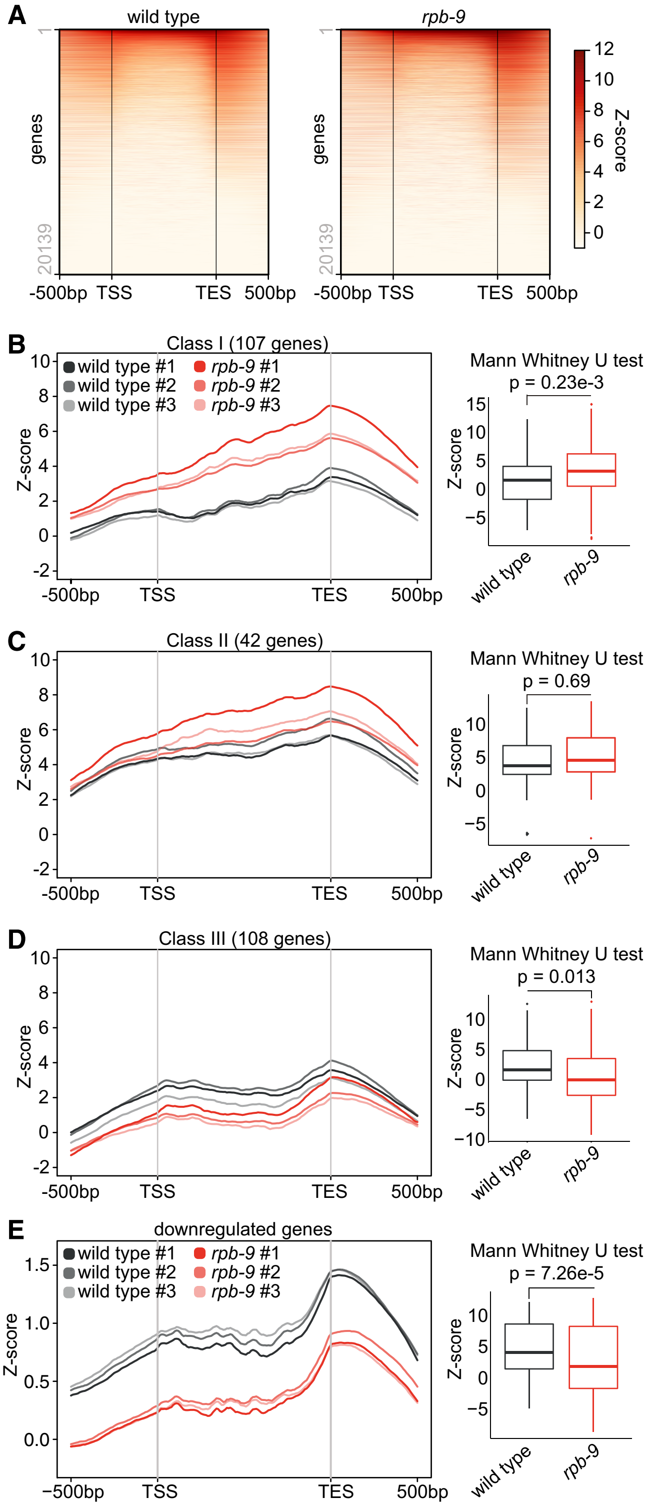


**Figure EV2. Related to Fig 4.**

A Analysis of differential RNA Pol II binding (RPB-1/AMA-1 ChIP-seq) over all genes in *rpb-9 (mj261)* mutants compared with wild type.

B–D Analysis of RNA Pol II binding at upregulated genes in *rpb-9 (mj261)* mutants, as defined in Appendix Fig S2B. Class I: upregulated genes with increased RNA Pol II binding (B); class II: upregulated genes with invariant RNA Pol II binding (C); class III: upregulated genes with reduced RNA Pol II binding (D). $n = 3$ biological replicates are shown per each genotype (RNA-sequencing filtering from total RNA "Ribo-Zero" RNA-seq libraries). Central bands represent the median, boxes represent the 25$^{th}$ and 75$^{th}$ percentiles, and whiskers represent the lowest and highest values, excluding outliers.

E Analysis of RNA Pol II binding at downregulated genes in *rpb-9 (mj261)* mutants ($n = 3$), as defined in Fig 3C.

© 2021 The Authors

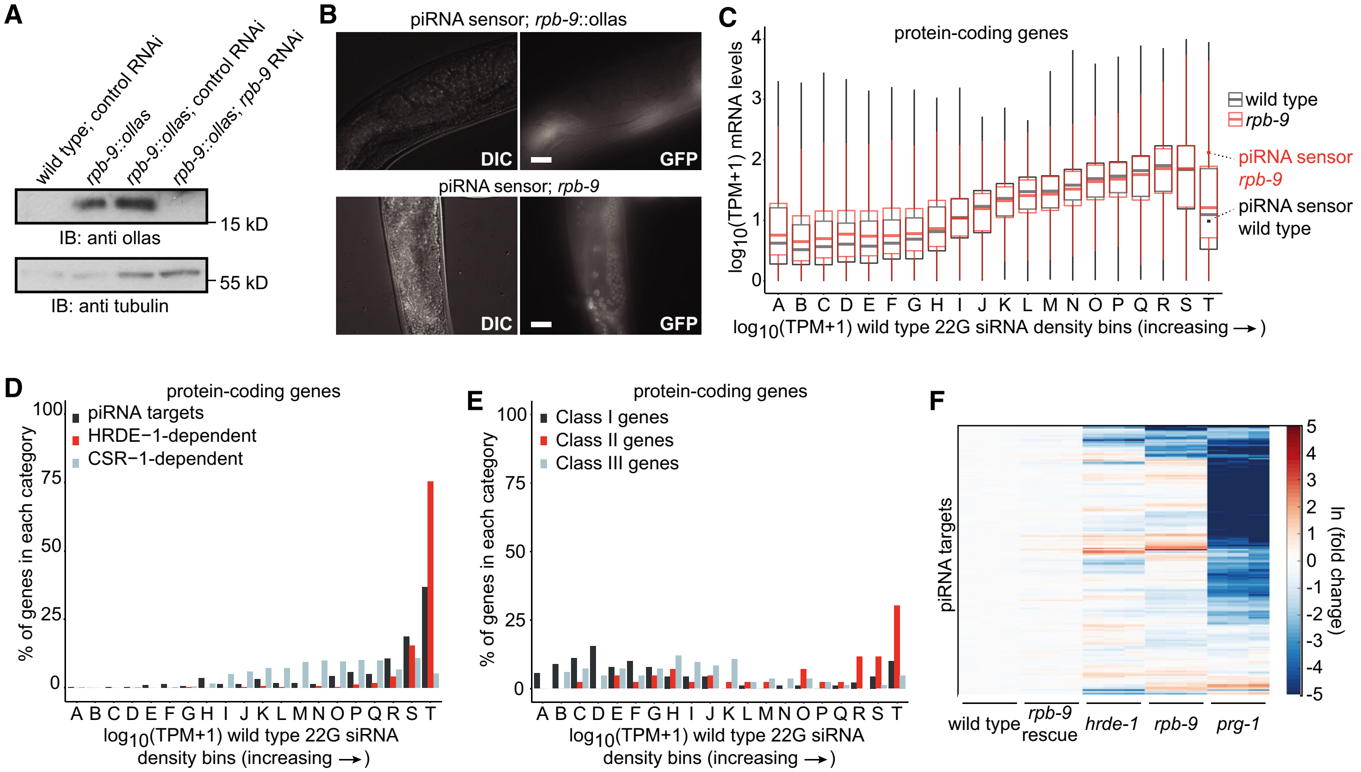


**Figure EV3. Related to Fig 5.**

A Western blot quantification of the amount of RPB-9 protein in the *rpb-9::ollas* line by Western blot. *rpb-9* RNAi samples are shown for control.

B Representative DIC and fluorescence microscopy images of piRNA sensor expression in *rpb-9::ollas (mj604)*. *rpb-9 (mj261)* is shown for control. Scale bar = 20 µm.

C Transcriptome binning according to increasing 22G siRNA density in wild-type animals (gray, $n = 3$). Mean normalized *rpb-9 (mj261)* mRNA reads (red, $n = 3$) are overlaid with mean normalized wild-type mRNA reads (gray) (total RNA Ribo-Zero RNA-seq libraries). The piRNA sensor transcript is highlighted in red *(rpb-9 (mj261))* and gray (wild type). Central bands represent the median, boxes represent the 25$^{th}$ and 75$^{th}$ percentiles, and whiskers represent the lowest and highest values, excluding outliers.

D Distribution of piRNA targets (as defined in Ref. (Bagijn *et al*, 2012) and of HDRE-1- and CSR-1-dependent 22G siRNAs across bins as defined in (C)).

E Distribution of class I, class II, and class III genes (as defined in Fig EV2C–E) across bins as defined in (C)).

F Cluster analysis of 22G siRNAs mapping to piRNA target genes in wild-type, *rpb-9 (mj261)*, *rpb-9* rescue *(mj261; mjSi70)*, *prg-1 (n4357)*, and *hrde-1 (tm1200)* animals (5′-independent small RNA libraries).

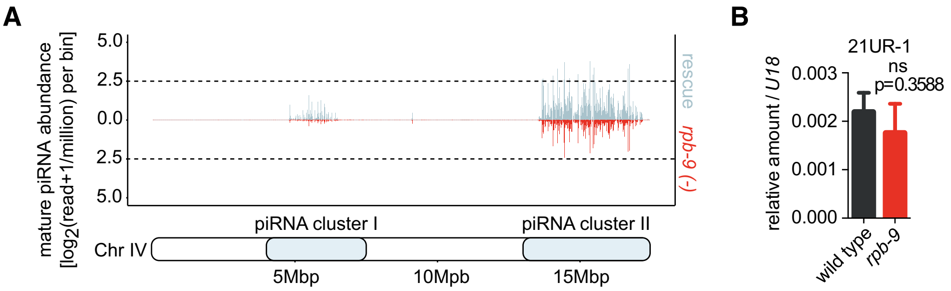


**Figure EV4. Related to Fig 6.**

A Mature piRNA expression along chromosome IV coordinates (motif-dependent piRNA clusters I and II) in *rpb-9 (mj261)* and *rpb-9* rescue *(mj261; mjSi70)* animals.

B RT–qPCR quantification of mature piRNA 21UR-1 levels in wild-type and *rpb-9 (mj261)* animals. $n = 3$ biological replicates, mean and SD shown (two-tailed *t*-test).

 © 2021 The Authors

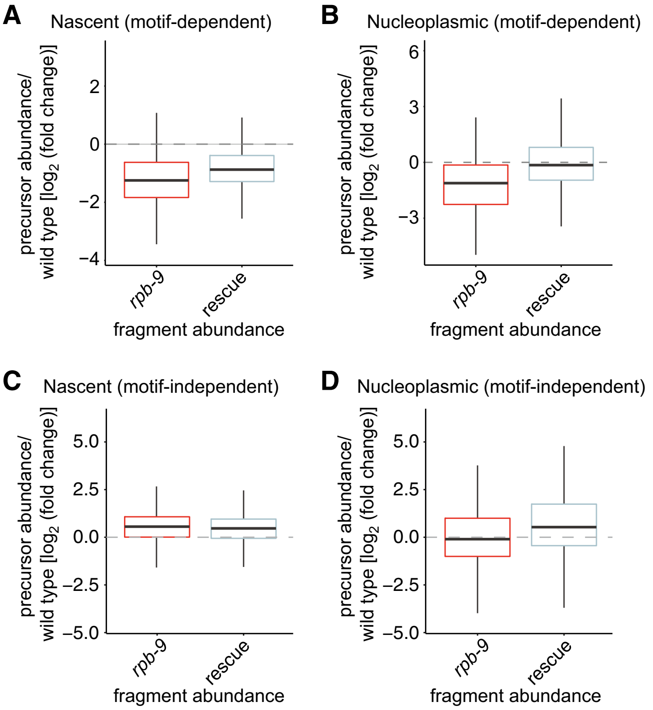


**Figure EV5. Related to Fig 7.**

A Motif-dependent nascent piRNA precursor abundance in *rpb-9* (*mj261*) and rescue *(mj261; mjSi70)* animals relative to wild type ($n$ = 2).

B Motif-dependent nucleoplasmic piRNA precursor abundance in *rpb-9* (*mj261*) and rescue *(mj261; mjSi70)* animals relative to wild type ($n$ = 2).

C Motif-independent nascent piRNA precursor abundance in *rpb-9* (*mj261*) and rescue *(mj261; mjSi70)* animals relative to wild type ($n$ = 2).

D Motif-independent nucleoplasmic piRNA precursor abundance in *rpb-9* (*mj261*) and rescue *(mj261; mjSi70)* animals relative to wild type ($n$ = 2). Central bands represent the median, boxes represent the 25$^{th}$ and 75$^{th}$ percentiles, and whiskers represent the lowest and highest values, excluding outliers.

© 2021 The Authors
