# Supplementary material for: The RNA polymerase II subunit RPB‐9 recruits the integrator complex to terminate Caenorhabditis elegans piRNA transcription
Source: EMBO J. 2021 Feb 3;40(5):e105565. doi: 10.15252/embj.2020105565 (PMC7917558; doi:10.15252/embj.2020105565)

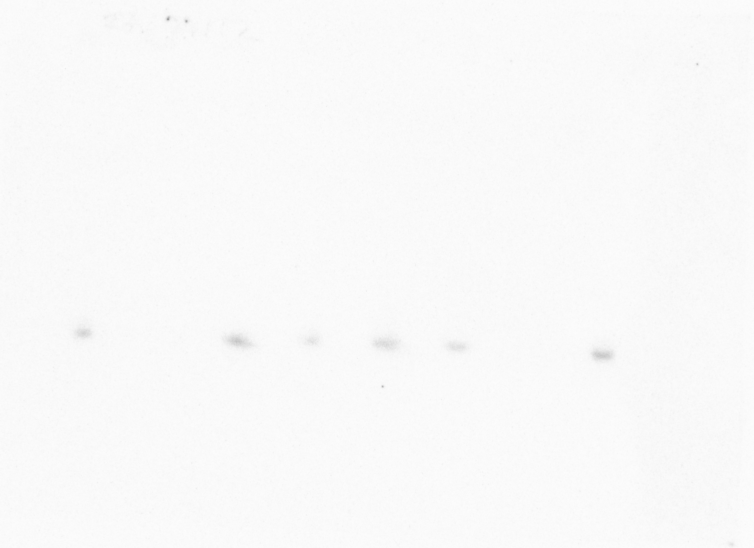

Supplement: Supplementary file 5 — Source Data for Figure 6 [file EMBJ-40-e105565-s003.pdf]
